# Health system implementation of clinical decision support to optimize respiratory pathogen profile utilization

**DOI:** 10.1128/jcm.01909-25

**Published:** 2026-07-20

**Authors:** Erika M. Flores, Yun Li, Jenna T. Reece, Mika Epps, Onyeka Nwankwo, Judith O'Donnell, Laurel Glaser, Kyle G. Rodino

**Affiliations:** 1 Department of Pathology and Laboratory Medicine, Hospital of the University of Pennsylvania 21798, Philadelphia, Pennsylvania, USA; 2 Department of Pathology and Laboratory Medicine, Children’s Hospital of Philadelphia, Philadelphia, Pennsylvania, USA; 3 Department of Biostatistics, Epidemiology, and Informatics, Perelman School of Medicine of the University of Pennsylvania 14640, Philadelphia, Pennsylvania, USA; 4 Pediatric IDEAS Research Group of Clinical Futures, Children’s Hospital of Philadelphia, Philadelphia, Pennsylvania, USA; 5 Department of Pediatrics, Perelman School of Medicine of the University of Pennsylvania 14640, Philadelphia, Pennsylvania, USA; 6 Penn Medicine, University of Pennsylvania Health System 6569, Philadelphia, Pennsylvania, USA; Cleveland Clinic, Cleveland, Ohio, USA

**Keywords:** respiratory pathogen profile, diagnostic stewardship, seasonal respiratory viruses, multiplex PCR

## Abstract

Overuse of respiratory pathogen profiles (RPPs) in immunocompetent patients offers limited clinical benefit and may contribute to unnecessary hospital costs. Evidence supports RPP use primarily in severely ill, immunosuppressed, or pediatric patients, while smaller, targeted tests are more appropriate for routine cases . The goal of this study was to evaluate the impact of a clinical decision support (CDS) intervention on RPP utilization and associated reagent costs. We reviewed RPP utilization across the health system and implemented a CDS tool with pre-approved clinical indications to guide appropriate RPP ordering or redirect testing to a smaller seasonal respiratory viral profile (SRVP). Initiation of an RPP order through the CDS tool presented the SRVP as the default, pre-selected option, with users prompted with specific built-in clinical indications before selecting whether to proceed with an RPP or SRVP. Data from inpatient and emergency department settings from January 2022 to January 2026 were used to assess RPP ordering practices pre- and post-CDS implementation. The impact of the CDS implementation on RPP orders was assessed using an interrupted time series analysis. Retrospective analysis revealed a year-to-year increase in RPP orders from 2022 to 2024. After CDS implementation, there was an immediate and significant decrease in RPPs compared to the pre-CDS period (−946.70, 95% CI: −1,320.2 to −573.2). This corresponded to $2.26 million in reagent cost savings. Overall, the introduction of the CDS successfully reduced the overutilization of large respiratory pathogen syndromic panels by encouraging the use of a SRVP when clinically appropriate.

**IMPORTANCE:**

Diagnostic stewardship ensures that multiplex molecular assays are used to deliver the right test, for the right patient, at the right time. Through coordinated input from interdisciplinary teams across the diagnostic pathway, stewardship enhances test selection, resource utilization, and interpretation of complex syndromic panels. Although multiplex PCR assays offer high sensitivity, their ability to detect residual nucleic acid from nonviable or colonizing organisms can create diagnostic ambiguity; therefore, it is important to have stewardship frameworks in place to guide appropriate test ordering, timing, and result interpretation and to avoid misinterpreting results. Through integration of laboratory expertise and evidence-based indications, diagnostic stewardship enhances the clinical value of multiplex testing and limits unnecessary treatment and downstream costs.

## INTRODUCTION

Respiratory pathogen profile panels are multiplex nucleic acid amplification tests (NAATs) assays that enable the simultaneous detection and differentiation of up to 22 respiratory pathogens, including viral and atypical bacterial targets. Currently, five major US FDA-approved respiratory multiplex assays are available, including the cobas eplex Respiratory Pathogen Panel 2 (Roche Diagnostics, Indianapolis, IN) and BioFire Respiratory 2.1 Panel (bioMérieux, Salt Lake City, UT), both utilized within the Penn Medicine System (1, 2). These assays offer several advantages: high sensitivity, rapid results, and support antibiotic stewardship by potentially providing clinicians with results to guide therapy decisions (3).

Despite these advantages, several drawbacks must be considered when implementing RPPs in routine practice. For example, although these assays demonstrate high analytical sensitivity, their clinical specificity is limited by the inability to differentiate between active infection and residual nucleic acid from prior infections (4, 5). Prolonged viral shedding after symptom resolution has been well documented, particularly in children and immunocompromised patients, with pathogens such as severe acute respiratory syndrome coronavirus 2 (SARS-CoV-2), influenza, rhinovirus, adenovirus, and human metapneumovirus (hMPV), raising the risk of misinterpreting clinically insignificant detections (6–12). Thus, these assays can lead to challenges in result interpretation, unnecessary infection prevention measures, or extended hospitalizations when organisms of unclear significance are detected. Moreover, in most instances, the detection of respiratory viruses in immunocompetent patients rarely alters clinical management, with the exception of influenza and SARS-CoV-2; therefore, routine RPP testing is not recommended in the outpatient setting (13–16). Instead, RPP testing is primarily reserved for immunocompromised patients given the significant morbidity and mortality associated with respiratory viral infections in this population (13). Taken together, these key points underscore the importance of diagnostic stewardship to ensure that RPP testing is applied selectively to patients for whom it is most beneficial.

The Penn Medicine Respiratory Pathogen Committee is comprised of emergency medicine, ambulatory, transplant, oncology, and infectious disease physicians, as well as hospital epidemiologists and laboratory directors. This team oversees seasonal respiratory virus testing across all hospitals in the health system. As part of this work, the committee reviewed RPP utilization over a two-and-a-half-year period with the goals of understanding system-wide practice and identifying variability in practice across sites, developing appropriate diagnostics stewardship interventions, and assessing the impact of those interventions post-implementation. The post-implementation objectives of this study were to quantify changes in test ordering following stewardship intervention and analyze reasons for bypassing testing recommendations to order RPPs.

## MATERIALS AND METHODS

### RPP testing

Respiratory pathogen profile testing was performed at three central laboratories, PennLab, Princeton, and Chester County Hospital. PennLab provides diagnostic services for the Hospital of the University of Pennsylvania (HUP), HUP–Cedar Avenue, Penn Presbyterian Medical Center, and Pennsylvania Hospital, together comprising a total of 1,931 staffed beds. Princeton Medical Center and Chester County Hospital, each with over 300 beds, are also part of the Penn Medicine Health System but perform diagnostic testing independently. These labs use either the cobas eplex Respiratory Pathogen Panel 2 (Roche Diagnostics, Indianapolis, IN) or the BioFire Respiratory 2.1 Panel (bioMérieux, Salt Lake City, UT). These assays were performed on one of the following specimens: nasopharyngeal swabs collected in saline or viral transport medium, bronchoalveolar lavage, or nasopharyngeal aspirates (collected from pediatric patients only).

### SRVP testing

Seasonal respiratory viral profile testing was performed using nasopharyngeal swabs in viral transport media on the Xpert Xpress CoV-2/Flu/RSV plus or Xpert Xpress SARS-CoV-2 (Cepheid, Sunnyvale, CA), for the qualitative detection and differentiation of SARS-CoV-2, influenza A/B, and respiratory syncytial virus. Testing with the SRVP was recommended for patients that did not meet the specified criteria in the CDS. The SRVP is aligned with local pathogen seasonality; SARS-CoV-2 is included year-round, while influenza A/B and respiratory syncytial virus (RSV) are added or removed based on seasonal activity. Influenza and RSV are removed from the SRVP in the Spring when local test positivity is less than 1% for 4 consecutive weeks and added in the Fall to align with predicted resurgence. This is driven off data from the Penn Medicine Respiratory Virus Weekly Summary Dashboard and approved for change by the Penn Medicine Respiratory Pathogen Committee. At the time of intervention, the SRVP consisted of four targets: influenza A/B, RSV, and SARS-CoV-2.

### Stewardship intervention

Specific guidelines for RPP testing at our institution were absent prior to CDS introduction. The Penn Medicine Respiratory Pathogen Committee established appropriate indications for RPP testing through review of current literature, professional society guidance, and consensus among committee members (13). The respiratory pathogen committee outlined indications for ordering an RPP to state that the use of this large panel should be limited to symptomatic patients who meet one or more of the following criteria: (i) active liquid oncology or stem cell transplantation, or receiving CAR T-cell therapy; (ii) will or have received solid organ transplants; (iii) critically ill children with an initial negative SRVP result; (iv) critically ill (ICU) with pneumonia; or (v) severe underlying respiratory condition.

**Fig 1:**
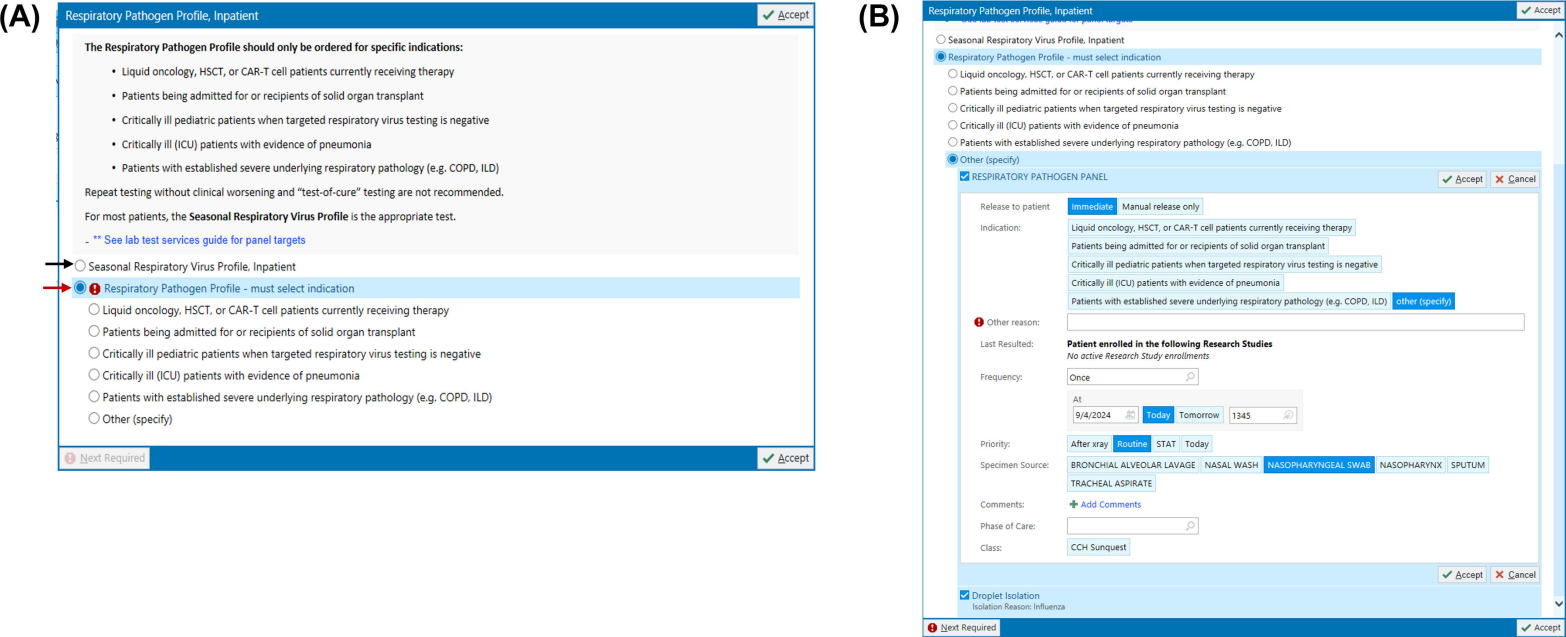
Example of a respiratory pathogen profile order panel with the integrated clinical decision support tool. The SRVP is presented as the default, pre-selected option when an RPP order is initiated through the CDS (black arrow). (**A**) The user is alerted with the specific indications for ordering an RPP and must then choose if they wish to proceed with a SRVP or an RPP (red arrow). If the user proceeds to order a RPP panel (highlighted in blue), then they must choose an option from the available radio buttons or select "Other" to list a custom reason for ordering the test. (**B**) Once the user has selected "Other," they are expected to specify the exact reason for ordering on the "Other reason" text-box. This panel is also showing additional information (i.e., time of order, specimen source) that the provider will select before placing the order.

A passive CDS tool, which presents the described clinical guidance without preventing order placement, was implemented in the EHR on 17 September 2024, for inpatient and emergency department settings across all six hospitals (Fig. 1). Upon opening the CDS tool, the SRVP is displayed as the default, pre-selected option for routine respiratory virus testing. The RPP is listed as a secondary option and requires providers to either select one of the predefined clinical indications aligned with testing recommendations via radio buttons or enter a custom indication under “Other” to proceed with ordering. This tool was designed with a soft stop that allows providers to place the order after documenting a free-text response, and there is no approval criteria to prevent orders from occurring.

The CDS tool was introduced through a coordinated rollout that included presentations to healthcare epidemiologists and emergency department physicians, along with distribution to key communicators and stakeholders accompanied by RPP utilization data. A memo detailing the introduction of a new RPP order panel with the integrated CDS tool was emailed to the Penn Medicine community 1 week prior to its introduction. This memo also provided a link to the Association for Diagnostics and Laboratory Medicine (ADLM) Guidance Document on Laboratory Diagnosis of Respiratory Viruses (13), which was, in part, used to establish appropriate testing guidelines, and an overview of how the CDS tool functions.

### RPP utilization review pre- and post-CDS implementation

A comprehensive data set of RPP orders was extracted from the PennChart electronic health record (EHR) system (© 2025 Epic Systems Corporation, Verona, WI), spanning January 2022 through January 2026, and included orders placed in both inpatient and emergency department settings across six Penn Medicine academic and community hospitals. The data set contained the following information: order date, patient facility, resulting laboratory, order name, patient medical record number, name, and specialty of authorizing provider. The number of RPP orders placed each month was quantified and analyzed to assess trends before and after CDS implementation using the extracted PennChart data.

### Assessing cost-savings estimates after CDS implementation

Reagent spend was evaluated using two approaches: a primary model estimating savings from predicted RPP orders with CDS vs counterfactual estimates (detailed in the Statistical Analyses section) and a secondary analysis comparing observed RPP orders to counterfactual estimates. The total of observed RPP orders from October 2024 to January 2026 was subtracted from the total of counterfactual RPP orders for the same period. Each RPP was estimated to cost between $100 and $120, and savings were calculated by multiplying this range by the change in order volumes.

### Quantification of SRVP orders and estimation of cost savings

A third EHR data pull was performed to compare the volume of SRVP orders placed in a 5-month period (November–March) in 2022 through 2025, which include periods before and after CDS implementation. Data sets were filtered to identify order sets containing Influenza A/B and RSV, as these pathogens are included in the SRVP. Inpatient and emergency department orders were subsequently quantified. The total number of orders was used to calculate the percentage increase for each season relative to the preceding one. The difference in SRVP volume was defined as the difference of the season after the CDS (November 2024–March 2025) relative to the prior season (November 2023–March 2024), and this difference was multiplied by $60 (the estimated cost of each SRVP panel). September and October were excluded from the analysis to align the months for which the SRVP was available across 2023 and 2024. SARS-CoV-2, Influenza, and RSV were offered at different times in 2023 and 2024, in November and September, respectively.

### Evaluating the summary of indications for ordering an RPP through the CDS tool

As mentioned above, ordering providers must select an indication from the available radio button options in the RPP order panel or choose “Other” to enter a custom reason for the test. The selected indications and free-text entries provided by ordering providers as justification for ordering an RPP test were extracted from PennChart and analyzed. These entries were classified into three categories: CDS defined (indication was consistent with the predefined radio-button clinical indications), free-text responses (responses specified under “Other” indications for ordering an RPP), and no indication for testing (the text box contained no interpretable entries). The free-text responses were further analyzed to distinguish appropriate clinical indication responses from those that did not have a clear clinical indication for ordering an RPP (Table S4, “Indications that do not align with the established clinical utility of RPPs”). Specific indications for RPP testing entered as free-text under the “Other” option and with clinical justification provided are summarized in Table 3. Additionally, the specific pathogens of concern listed in the “Other” prompt were also counted and listed in Table S3.

### Statistical analyses

An interrupted time series analysis was used to evaluate the immediate and longer-term impact of implementing the CDS tool on monthly RPP order volume. The outcome was the number of RPP orders aggregated by calendar month from January 2022 through January 2026. September 2024 was designated as the intervention month, with October 2024 corresponding to the first full month after CDS implementation.

A segmented linear regression model was fit with terms for (i) baseline time (months since January 2022), (ii), an indicator for the post-intervention period to estimate the immediate level change at CDS initiation, and (iii) interaction between time and the post-intervention indicator to estimate any change in slope after CDS initiation. The immediate effect was quantified as the estimated change in the monthly number of RPP orders in September 2024 compared with the counterfactual projected from the pre-intervention trend, and the longer-term effect was quantified as the post-intervention monthly trend (slope) and the difference between pre- and post-intervention slopes. The counterfactual number of RPP orders was estimated by extrapolating the pre-CDS trends into the post-CDS period, representing expected orders in the absence of the CDS. Differences in RPP orders were calculated by subtracting the counterfactual predictions from the predicted RPP orders.

Newey–West standard errors were used to account for serial correlation in monthly observations. The autocorrelation lag length was set to 2 months, estimated by the Cumby–Huizinga general test. Statistical significance was assessed using two-sided tests with *α* = 0.05. Analyses were conducted using Stata itsa package (Linden, the Stata Journal, 2015; StataCorp, College Station, TX), and the figure was created using R (version 4.4.3. Vienna, Austria) (17).

## RESULTS

**Fig 2:**
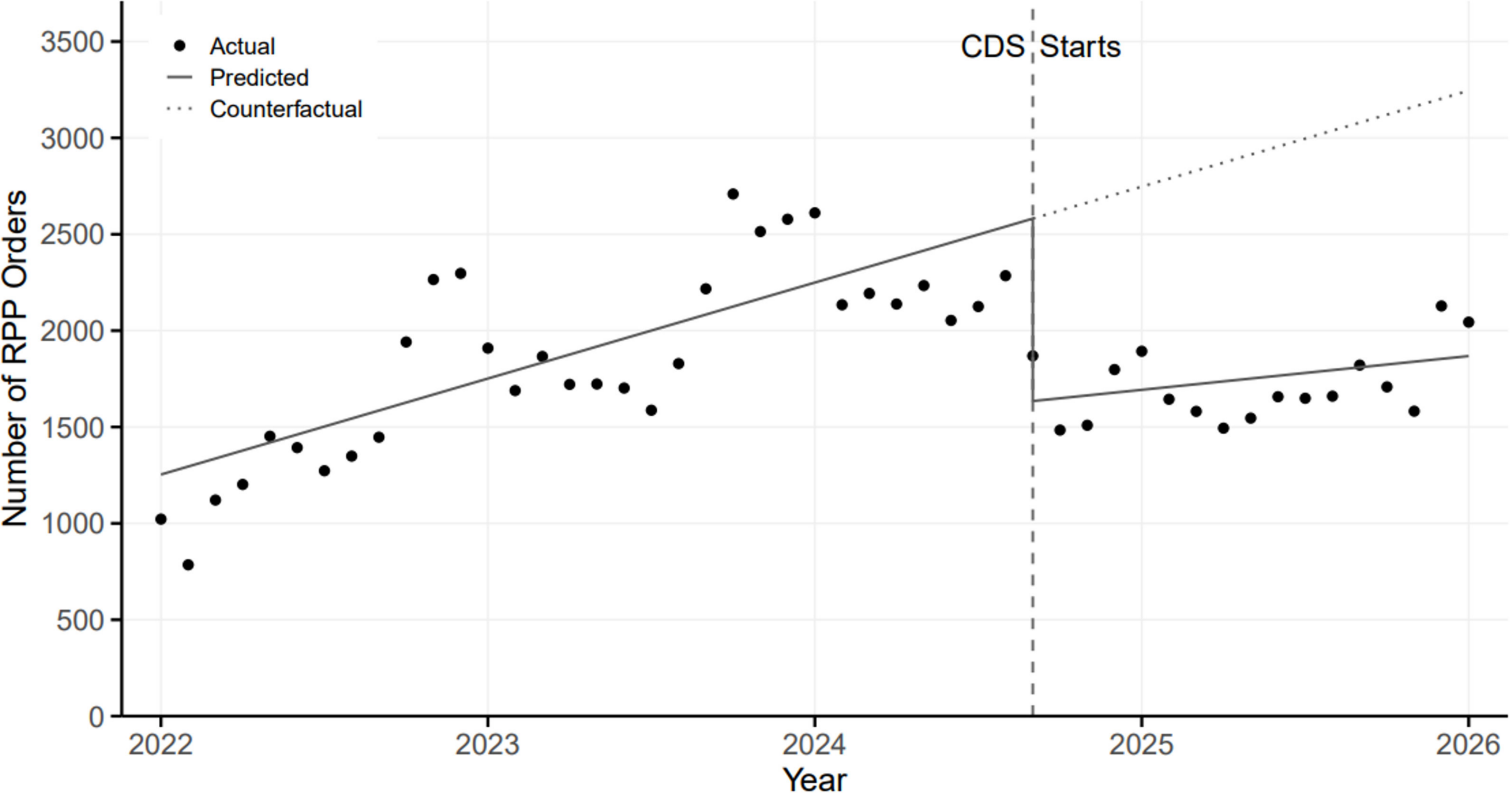
Interrupted time series of monthly RPP orders before and after CDS implementation. Points show observed monthly RPP orders from January 2022 through January 2026. The vertical dashed line indicates CDS implementation (September 2024). Solid and dotted lines show model-predicted trends estimated from segmented linear regression.

Interrupted time series analysis of monthly RPP orders showed a significant upward trend pre-CDS of 41.5 additional orders per month (95% CI: 25.7 to 57.3) from January 2022 to August 2024 (Fig. 2; Table 1). This increase was not driven by changes in inpatient admissions or ED visits (Fig. S1). Given the significant upward trend, combined with observed variability in test utilization across similar domains within the health system (e.g., emergency department and inpatient practice; data not shown), development and implementation of an integrated CDS tool were pursued.

**TABLE 1:** Level and trend changes in monthly RPP orders before and after CDS implementation using Interrupted Time Series Regression^*a*^

Effect	Estimate	95% CI	*P*-value
Pre-CDS monthly rate of change in the number of RPP orders	41.49	[25.73, 57.26]	<0.001
Immediate change in the number of orders after CDS start	−946.70	[−1,320.19, −573.21]	<0.001
Post-CDS monthly rate of change in the number of RPP orders	14.60	[−4.02, 33.21]	0.124
Difference between post- and pre-CDS implementation in the rate of change in the orders	−26.90	[−50.12, −3.68]	0.023

^*a*^ January 2022–August 2024, September 2024, and October 2024–January 2026 correspond to the pre-CDS, implementation, and post-CDS periods, respectively.

Following the introduction of testing guidelines and the CDS tool in September 2024, there was an immediate and significant decrease of 946.7 orders relative to the counterfactual predicted from the pre-CDS trend (95% CI: −1,320.2 to −573.2). After implementation, the monthly RPP order change shows a modest upward trend, 14.6 orders per month (95% CI: −4.0 to 33.2), which was not statistically significant. Compared to the pre-CDS trend, this represents a 26.9 orders per month decrease (slope change: −26.9; 95% CI: −50.1 to −3.7) (Fig. 2; Table 1). The primary cost savings model showed that CDS implementation was associated with ~18,800 fewer RPP orders compared to expected use without CDS, corresponding to an estimated cost savings of ~$1.88 million to ~$2.26 million over the study period (Table 2). The second approach showed a reduction in ~19,745 RPP tests compared to the counterfactual numbers, with cost savings of ~$1.97 million to ~$2.37 million (Table S1).

**TABLE 2:** Monthly counterfactual and CDS-predicted RPP orders and associated reagent cost savings^*a*^

Month and year	Time (*t*)	Predicted with CDS	Counterfactual	Difference
October 2024	34	1,649.15	2,622.75	−973.60
November 2024	35	1,663.74	2,664.24	−1,000.50
December 2024	36	1,678.34	2,705.73	−1,027.39
January 2025	37	1,692.93	2,747.23	−1,054.29
February 2025	38	1,707.53	2,788.72	−1,081.19
March 2025	39	1,722.13	2,830.21	−1,108.09
April 2025	40	1,736.72	2,871.70	−1,134.98
May 2025	41	1,751.32	2,913.20	−1,161.88
June 2025	42	1,765.91	2,954.69	−1,188.78
July 2025	43	1,780.51	2,996.18	−1,215.68
August 2025	44	1,795.10	3,037.68	−1,242.57
September 2025	45	1,809.70	3,079.17	−1,269.47
October 2025	46	1,824.29	3,120.66	−1,296.37
November 2025	47	1,838.89	3,162.16	−1,323.27
December 2025	48	1,853.49	3,203.65	−1,350.16
January 2026	49	1,868.08	3,245.14	−1,377.06
Total		28,137.83	46,943.11	−18,805.27
Total cost savings				$1,880,527− $2,256,632.40

^*a*^ The predicted RPP orders with CDS in place is compared to counterfactual estimates representing expected number of orders without CDS. The difference is calculated as predicted RPP orders minus counterfactual. The total cost savings was estimated by multiplying the total difference times the estimated cost of each RPP. Time represents the number of months after January 2022.

Despite the ability to reroute ordering to the SRVP, implementation of the CDS tool was not associated with substantially increased SRVP use. After CDS introduction (November 2024 to March 2025), SRVP orders increased by approximately 16% compared to the prior season (November 2023 to March 2024) (Table S2). This 16% increase is in line with historical annual test volume increase and represents less annual growth than that of the previous respiratory seasons.

**Fig 3:**
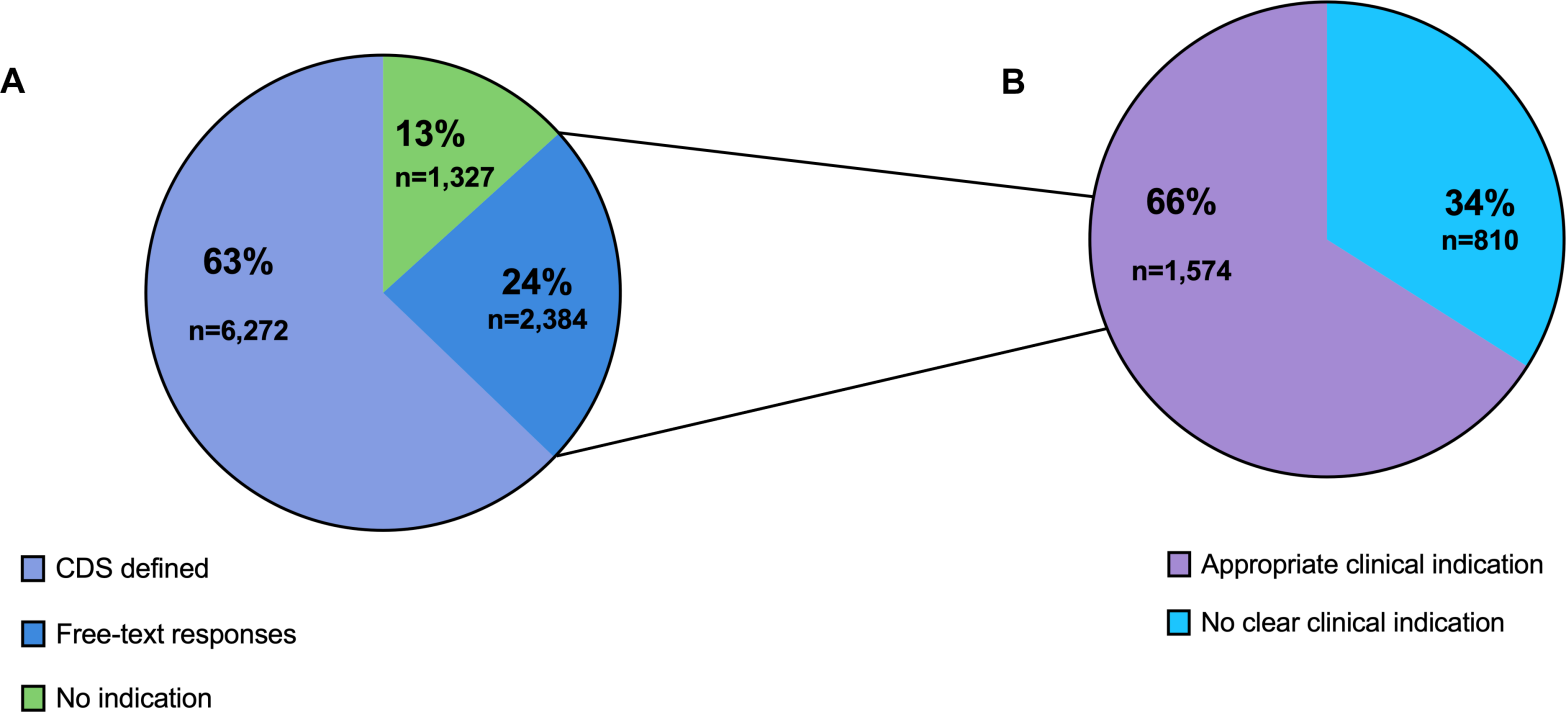
Summary of testing indications for ordering an RPP through the CDS panel. (**A**) CDS-defined indications were those that were consistent with the established RPP testing guidelines displayed as radio buttons within the CDS. Free-text responses refer to answers that were typed into the free-text box under "Other" field when providers specified reasons for ordering a RPP. Some responses contained no meaningful text, and these were classified as having no indication. (**B**) Of the 2,384 free-text responses, 66% provided an appropriate clinical indication ordering a RPP and 34% lacked clear clinical indication. Over the course of 17 September 2024–31 March 2025, there was a total of 9,983 RPP tests ordered.

Following CDS implementation, reasons for RPP ordering were categorized as CDS-defined, free-text, or no indication. Most orders (63%) used approved CDS indications, 24% used free-text, and 13% no indication provided (likely add-on orders that do not utilize the CDS panel and, therefore, do not require an indication) (Fig. 3A). Among 2,384 free-text entries, 66% included appropriate clinical indications, while 34% did not provide clear clinical indications for RPP use (Fig. 3B). Common free-text responses used to order an RPP included patient with respiratory symptoms without underlying chronic disease (72%), suspicion for advanced lung disease (12.8%), and concern for specific pathogens (11%). Less common reasons included reflex testing after negative SRVP, asthma exacerbation, lack of improvement after antibiotics, and transfer-related testing (Table 3). The most frequently cited pathogen-specific reason for ordering RPP was *Mycoplasma pneumoniae* (80%), followed by *Bordetella pertussis* (14%) (Table S3).

**TABLE 3:** Frequent reasons listed in the “other” free-text box used for ordering a RPP panel^*a*^

Reasons	No. of orders	Percent of orders
Symptoms of respiratory tract infection in patients without specified underlying disease	1,137	72.2%
Signs of advanced lung disease	201	12.8%
Concern for a particular respiratory infection	174	11.1%
Negative SRVP	26	1.7%
Asthma exacerbation	19	1.2%
Failure of symptom improvement after administrations of antibiotics	13	0.8%
Facility transfer	4	0.3%
**Total**	**1,574**	

^*a*^ These were the responses recorded to place a RPP order using indications that were not consistent with the CDS-defined recommendations.

## DISCUSSION

In response to year-over-year increase in RPP utilization, a CDS tool was integrated into the EHR to provide clinicians with evidence-based recommendations regarding RPP test ordering. The interrupted time series analysis findings showed a large immediate downward shift in monthly RPP orders after CDS implementation, along with a significant reduction in the month-to-month growth rate compared with the pre-intervention period (14.60 vs 41.49; −26.90). The post-CDS monthly trend in RPP orders (14.6) was not statistically significant, indicating that while there is a gradual growth in overall use, the CDS tool’s reduction in ordering is largely being maintained. The timing of the intervention likely yields conservative estimates, as CDS was implemented mid-month in September 2024, which was designated as the intervention month in the interrupted time series analysis. The estimated immediate level change is likely biased toward the null because this month included partial exposure. Thus, the true immediate impact of CDS on RPP ordering is likely greater than estimated. Consistent with this, a ~$112,000 difference in estimated savings across two different modeling approaches further supports that CDS effects may be underestimated.

Although interrupted time series designs are vulnerable to co-interventions or other contemporaneous changes, we are not aware of system- or practice-level factors that would explain the observed decline in RPP orders. To our knowledge, no significant changes in ED and inpatient admissions have occurred, suggesting that the reduction in RPP ordering was a result of provider behavior driven by the CDS tool rather than changes in patient volume. Notably, retrospective analysis of re-routing data demonstrated that approximately 30% of providers redirected orders to the SRVP rather than proceeding with an RPP (data not shown). However, we did not observe an equivalent increase in the SRVP tests after introducing the CDS. In contrast, increase in SRVP orders post-CDS was in line with historical trends. We hypothesize that providers were previously ordering both SRVP and RPP, with the CDS re-routing resulting in ordering of only the SRVP, effectively decreasing RPP test volume and reducing redundant testing. Continued monitoring of SRVP orders was limited because testing is seasonal and was tracked using influenza/RSV as surrogates; however, these targets are not routinely performed in the off-season, thus making accurate assessment outside peak respiratory virus months challenging.

Further reinforcing the need for RPP stewardship, the Centers for Medicare & Medicaid Services (CMS) limits outpatient testing to cases where results are expected to guide management and improve outcomes. Accordingly, CMS only deems small panels (≤5 pathogens) medically necessary for outpatient claims, restricting reimbursement for larger panels (18). CMS policies reflect evidence showing limited and inconsistent clinical benefit from large syndromic panels. A systematic review of 15 studies found that rapid molecular tests did not reduce antibiotic prescribing, treatment duration, or hospital admissions though the data suggested that these assays may shorten hospital stays (19). In contrast, a study involving 191 patients showed a nonsignificant reduction in antibiotic use with RPP testing and no effect on antiviral prescribing or ED length of stay (20). Collectively, current evidence supports restricting RPP testing to immunocompromised patients, who are at higher risk for severe disease from common and opportunistic pathogens (13, 21). Although the data presented here include only inpatient and emergency department encounters, the CDS has since been expanded to outpatient settings. Outpatient data were not included in this study due to later integration.

A common free-text justification from ordering providers was concern for a specific pathogen, either named explicitly or inferred from associated symptoms (Table 3). Examples included viral exanthems or suspected viral myocarditis and interpreted as concern for enterovirus or other respiratory viruses (22–25); however, RPP testing is not useful for enterovirus myocarditis, which requires detection in CSF, blood, or cardiac tissue (23, 24, 26). Twenty-six RPP orders were placed after a negative SRVP despite CDS restrictions to critically ill patients, with no documented justification; however, patient-level linkage to re-routing data was unavailable, limiting further assessment (Table 3). Additional inappropriate orders included non-respiratory indications, pathogens not included in the panel, or vague justifications (Table S4). A separate “missing indication” category captured orders with no meaningful rationale (e.g., symbols entered to bypass CDS).

*M. pneumoniae* and *B. pertussis* were the two most common pathogen-specific indications provided as justification for RPP ordering. This is expected, as there was a peak of *M. pneumoniae* activity in the Fall of 2024 (27). Additionally, some health-system sites have pediatric emergency departments and inpatient services, making them more likely to need testing for *B. pertussis*. Within the Penn Medicine system, singleplex *M. pneumoniae* and *B. pertussis* PCR assays are not available in-house, and send-out testing is associated with prolonged turnaround times. Thus, use of the multiplex panel to access rapid testing for these pathogens is understandable. The Respiratory Pathogen Committee is continuing to monitor the frequency of these pathogen-specific justifications as a factor in evaluating the potential need for additional singleplex test offering.

The large data sets used to evaluate RPP ordering trends included hospital identifiers, allowing comparison across sites. Sorting by ordering indications revealed that some hospitals more effectively adopted the stewardship process than others. Specifically, hospitals with dedicated teams to promote the CDS were more likely to order panels for predefined indications, whereas hospitals without such advocacy demonstrated substantially higher rates of inappropriate or missing indications for RPP ordering. Notably, physician-leaders involved in CDS implementation actively encouraged appropriate diagnostic stewardship within their teams. No clinician concerns were identified, as evidenced by the absence of complaints through the safety monitoring process. These findings reinforce the importance of laboratory-clinical collaboration in designing CDS tools.

In summary, the CDS tool effectively improved diagnostic stewardship, resulting in an immediate and sustained reduction in RPP orders and associated costs. While its passive design allowed providers to bypass recommendations, the majority of RPP ordering was in line with institutional guidance. Further refinement of the CDS tool or alternative diagnostic stewardship strategies could be pursued to address the remaining areas for improvement. Overall, these findings highlight the value of targeted CDS interventions in promoting evidence-based diagnostic practice and improving patient care.

## SUPPLEMENTAL MATERIAL

Figure S1 jcm.01909-25-s0001.docx Number of inpatient and emergency department encounters.

Table S1 jcm.01909-25-s0002.docx Number of actual RPP orders that were used to estimate cost savings.

Table S2 jcm.01909-25-s0003.docx SRVP orders across three respiratory virus seasons.

Table S3 jcm.01909-25-s0004.docx Free-text responses of pathogens of interest to clinicians.

Table S4 jcm.01909-25-s0005.xlsx Described the free-text responses mentioned in the manuscript.

ASM does not own the copyrights to Supplemental Material that may be linked to, or accessed through, an article. The authors have granted ASM a non-exclusive, world-wide license to publish the Supplemental Material files. Please contact the corresponding author directly for reuse.
