# Supplementary material for: Health system implementation of clinical decision support to optimize respiratory pathogen profile utilization
Source: J Clin Microbiol. 2026 Jul 20;64(8):e01909-25. doi: 10.1128/jcm.01909-25 (PMC13463821; doi:10.1128/jcm.01909-25)
Supplement: Supplementary file 1 [file jcm.01909-25-s0001.docx]

**Supplementary Figure 1. Number of (A) inpatient and (B) emergency department encounters from January 2022- September 2024.**
