# Supplementary material for: Health system implementation of clinical decision support to optimize respiratory pathogen profile utilization
Source: J Clin Microbiol. 2026 Jul 20;64(8):e01909-25. doi: 10.1128/jcm.01909-25 (PMC13463821; doi:10.1128/jcm.01909-25)
Supplement: Supplementary file 2 [file jcm.01909-25-s0002.docx]

**Supplementary Table 1. Total number of observed RPP orders and estimated savings.** The total of counterfactual RPP orders was acquired from Table 2. The difference between observed and counterfactual order was −19,745.11 and this was multiplied times estimated cost of each RPP to estimate savings.
