# Supplementary material for: Health system implementation of clinical decision support to optimize respiratory pathogen profile utilization
Source: J Clin Microbiol. 2026 Jul 20;64(8):e01909-25. doi: 10.1128/jcm.01909-25 (PMC13463821; doi:10.1128/jcm.01909-25)
Supplement: Supplementary file 3 [file jcm.01909-25-s0003.docx]

**Supplementary Table 2. SRVP orders across three respiratory virus seasons and estimated savings after CDS introduction.** The number of SRVP orders from each season (November 2022–March 2023, November 2023–March 2024, and November 2024–March 2025) are shown. Difference in SRVP volume is defined as the difference of the season after the CDS (Nov 2024–Mar 2025) relative to the prior season (Nov 2023–Mar 2024).
